# Evaluating strategies for control of tuberculosis in prisons and prevention of spillover into communities: An observational and modeling study from Brazil

**DOI:** 10.1371/journal.pmed.1002737

**Published:** 2019-01-24

**Authors:** Tarub S. Mabud, Maria de Lourdes Delgado Alves, Albert I. Ko, Sanjay Basu, Katharine S. Walter, Ted Cohen, Barun Mathema, Caroline Colijn, Everton Lemos, Julio Croda, Jason R. Andrews

**Affiliations:** 1 Department of Medicine, Stanford University School of Medicine, Stanford, California, United States of America; 2 Agência Estadual de Administração do Sistema Penitenciário, Campo Grande, Brazil; 3 Department of Epidemiology of Microbial Diseases, Yale School of Public Health, New Haven, Connecticut, United States of America; 4 Department of Epidemiology, Columbia University Mailman School of Public Health, New York, New York, United States of America; 5 Department of Mathematics, Imperial College London, London, United Kingdom; 6 Department of Mathematics, Simon Fraser University, Burnaby, Canada; 7 School of Medicine, Federal University of Mato Grosso do Sul, Campo Grande, Brazil; 8 Oswaldo Cruz Foundation, Campo Grande, Brazil; University of California San Francisco, UNITED STATES

## Abstract

**Background:**

It has been hypothesized that prisons serve as amplifiers of general tuberculosis (TB) epidemics, but there is a paucity of data on this phenomenon and the potential population-level effects of prison-focused interventions. This study (1) quantifies the TB risk for prisoners as they traverse incarceration and release, (2) mathematically models the impact of prison-based interventions on TB burden in the general population, and (3) generalizes this model to a wide range of epidemiological contexts.

**Methods and findings:**

We obtained individual-level incarceration data for all inmates (*n* = 42,925) and all reported TB cases (*n* = 5,643) in the Brazilian state of Mato Grosso do Sul from 2007 through 2013. We matched individuals between prisoner and TB databases and estimated the incidence of TB from the time of incarceration and the time of prison release using Cox proportional hazards models. We identified 130 new TB cases diagnosed during incarceration and 170 among individuals released from prison. During imprisonment, TB rates increased from 111 cases per 100,000 person-years at entry to a maximum of 1,303 per 100,000 person-years at 5.2 years. At release, TB incidence was 229 per 100,000 person-years, which declined to 42 per 100,000 person-years (the average TB incidence in Brazil) after 7 years. We used these data to populate a compartmental model of TB transmission and incarceration to evaluate the effects of various prison-based interventions on the incidence of TB among prisoners and the general population. Annual mass TB screening within Brazilian prisons would reduce TB incidence in prisons by 47.4% (95% Bayesian credible interval [BCI], 44.4%–52.5%) and in the general population by 19.4% (95% BCI 17.9%–24.2%). A generalized model demonstrates that prison-based interventions would have maximum effectiveness in reducing community incidence in populations with a high concentration of TB in prisons and greater degrees of mixing between ex-prisoners and community members. Study limitations include our focus on a single Brazilian state and our retrospective use of administrative databases.

**Conclusions:**

Our findings suggest that the prison environment, more so than the prison population itself, drives TB incidence, and targeted interventions within prisons could have a substantial effect on the broader TB epidemic.

## Abstract 2

From a large cohort study in Brazil, Tarub Mabud and colleagues reveal that the prison environment, rather than inmates themselves, drives up TB infection rates that spill over into communities.

## Author summary

**Why was this study done?:**

- Tuberculosis (TB) remains a leading cause of infectious disease mortality worldwide, despite global efforts to combat it.
- Certain environments such as prisons predispose individuals to higher than normal risk of TB.
- High-risk prison environments may sustain and amplify TB transmission in their surrounding communities as individuals are incarcerated and released.
- The time course of TB risk following incarceration and release has not been well characterized, and the impact of interventions conducted in prisons upon the community TB epidemic is not known.

**What did the researchers do and find?:**

- We used Brazilian TB and prisoner databases to quantify the TB risk for individuals throughout incarceration and release from prison.
- We created a mathematical model to simulate prison-based TB interventions among prisoners, ex-prisoners, and the general population in a Brazilian community and then generalized this model to a wide range of epidemiological contexts.
- After incarceration, the TB risk for a prisoner rises to 30 times that of a community member, and this risk remains elevated for 7 years after release from prison.
- A comprehensive intervention including mass screening, entry and exit screening, improved diagnostics, and isoniazid preventive therapy conducted within Brazilian prisons would reduce active TB incidence by 79% in the prisons and by 40% in the general population.
- Across global contexts, prison-based TB interventions would have maximum effectiveness in reducing community incidence of TB in populations with a high concentration of disease in prisons and greater degrees of mixing between ex-prisoners and community members.

**What do these findings mean?:**

- The prison environment, more so than an inherently high-risk prison population, drives TB incidence in prisons.
- Targeted interventions within prisons could substantially impact TB epidemics in the general population.

## Introduction

The World Health Organization’s (WHO) “End Tuberculosis Strategy” targets a 90% reduction in tuberculosis (TB) incidence by 2035, which requires significant acceleration in the 2% annual decline seen at present [1]. A major obstacle to achieving TB control in low- and middle-income countries (LMICs) is thought to be the concentration of disease in high-burden subpopulations, which serve as reservoirs or amplifiers for TB epidemics in the general population. This concept is supported by some ecological analyses [2], but there have not been detailed epidemiologic investigations of “institutional amplifiers” or how targeting them might impact population-wide TB dynamics.

Prisons are a plausible candidate “institutional amplifier” for TB, globally. A recent meta-analysis estimated a median TB incidence rate ratio of 26 in prisons compared with the reference general population [3,4]. Genotypic evidence has supported linkage between TB cases in prisons and the general population [5,6], but these data have come from focal outbreaks and have not clarified the extent to which spillover from prisons may drive community epidemics [6–11]. Data on TB within correctional facilities are frequently not made available to the public, and there is a paucity of data on TB incidence following release from prison, hindering quantitative investigations on the dynamics of TB epidemics in prisons and the general population. This lack of data also impedes the development of interventions to combat TB. Most prisons in LMICs rely upon passive case detection, in which inmates are referred for diagnostic workup only after they become symptomatic. WHO conditionally recommends active case detection, including screening upon entry into prisons, but characterizes the supporting evidence as very low quality [12]. Consequently, few countries routinely perform enhanced TB control activities in prisons, such as entry and exit screening, periodic mass screening, or provision of isoniazid preventive therapy (IPT). There is a clear need for more data to guide the design and selection of interventions to combat the spread of TB in prisons and their surrounding communities.

To address these knowledge gaps, we utilized a unique opportunity to link comprehensive incarceration and TB notification datasets in the Brazilian state with the highest incarceration rate, Mato Grosso do Sul. We estimated hazards of TB during incarceration and upon release from prison, and we used these data to parameterize a mathematical model that describes the transmission dynamics of TB in prisons and the general population. We then projected the impact of interventions conducted within the prisons on TB incidence in the general population, further generalizing this to settings with varying incarceration rates and concentrations of TB within prisons.

## Methods

### Study setting

Brazil has the third-largest incarcerated population in the world [13], and a rapidly growing proportion of TB cases occur in the correctional system [5,14–16]. With a population of 2.6 million, the state of Mato Grosso do Sul lies in central-west Brazil, adjoining Paraguay and Bolivia. The state has the highest incarceration rate in Brazil (475 prisoners per 100,000 individuals), driven primarily by drug trafficking across country borders [17].

### Data collection

We determined the analysis plan prior to the capture of any data and followed this plan without major adjustments. To calculate the annual incidence of active TB among prisoners and ex-prisoners in Mato Grosso do Sul, we used two electronic databases: Sistema de Informação de Agravos de Notificação (SINAN, 2006–2015), the Brazilian Ministry of Health’s mandatory reporting system for TB; and Sistema Integrado de Gestão Operacional (SIGO, 2005–2014), the prison record system for Mato Grosso do Sul. We limited our analysis to the period between January 1, 2007, and December 31, 2013, for which complete data were obtained from both databases.

SINAN is populated with mandatory reporting forms that are filled out by healthcare providers on the day a TB diagnosis is determined. Submitted data comprise a wide range of demographic and clinical information including name, date of birth, date of death, date of TB diagnosis, and mother’s name. We had access to SINAN data only for the state of Mato Grosso do Sul.

SIGO contains the prison records of all individuals incarcerated in Mato Grosso do Sul. We received permission from the state prison administration agency to access SIGO within their prison computer network. In general, information can only be accessed for one prisoner at a time in SIGO, and only if the prisoner’s name or prison ID number are known; exportation of the entire database was not feasible given the high sensitivity of the data and the need to rigorously maintain confidentiality. We therefore obtained an autogenerated report from the SIGO platform, available to certain administrators, which described comprehensive prisoner movement within the state’s prisons and contained prisoner name, a unique identifier, and all dates of entry into, transfer between, and release from prison, along with corresponding prison names.

### Database linkage

**Fig 1:**
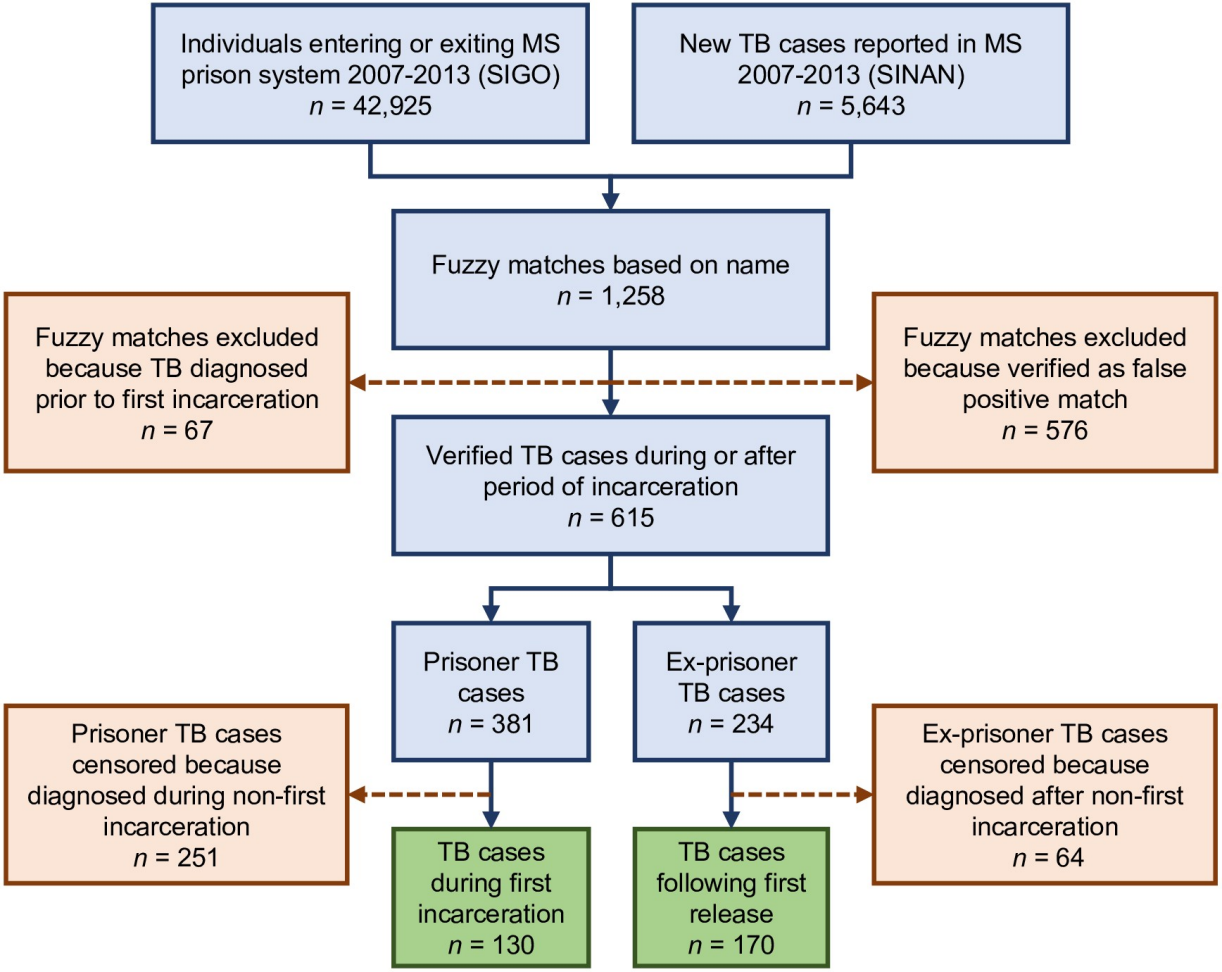
Flow diagram of database linkage and selection of individuals for survival analyses. MS, Mato Grosso do Sul; SIGO, Sistema Integrado de Gestão Operacional; SINAN, Sistema de Informação de Agravos de Notificação; TB, tuberculosis.

We identified prisoners and ex-prisoners who had been diagnosed with active TB between 2007 and 2013 by matching individuals in SINAN and the SIGO report (Fig 1). We limited SINAN to new adult active TB diagnoses (as opposed to recurrent cases) in Mato Grosso do Sul between the years 2007 and 2013 (*n* = 5,643). To link the two databases, we constructed a name-matching algorithm in GREL using OpenRefine version 2.6 [18], which combines phonetic fingerprinting and a distance function between strings, to identify “fuzzy” matches—names that are similar but not necessarily identical—between SINAN and the SIGO report [19,20]. We increased the allowable distance between matched names until we achieved a sensitivity of 100% when comparing the algorithm matches to an independent dataset of 42 confirmed active prisoner TB cases in the city of Dourados, Brazil (within Mato Grosso do Sul), in 2013.

The matching process produced 1,258 fuzzy matches between the two databases. Two investigators independently queried each fuzzy match within the official SIGO database, which allowed for manual comparison of date of birth, mother’s name, city of birth, and father’s name between SINAN and SIGO. From the pool of fuzzy matches, we verified 615 correctly matched individuals who were diagnosed with active TB either during or after a period of incarceration.

### Hazard estimation

We performed separate survival analyses for prisoners and ex-prisoners with the primary endpoint of TB diagnosis. We focused our analyses on first incarcerations and the release period following first incarceration, generating two cohorts: (1) prisoners who had their first incarceration between 2007 and 2013 and (2) ex-prisoners who had been released from their first incarceration between 2007 and 2013.

**Fig 2:**
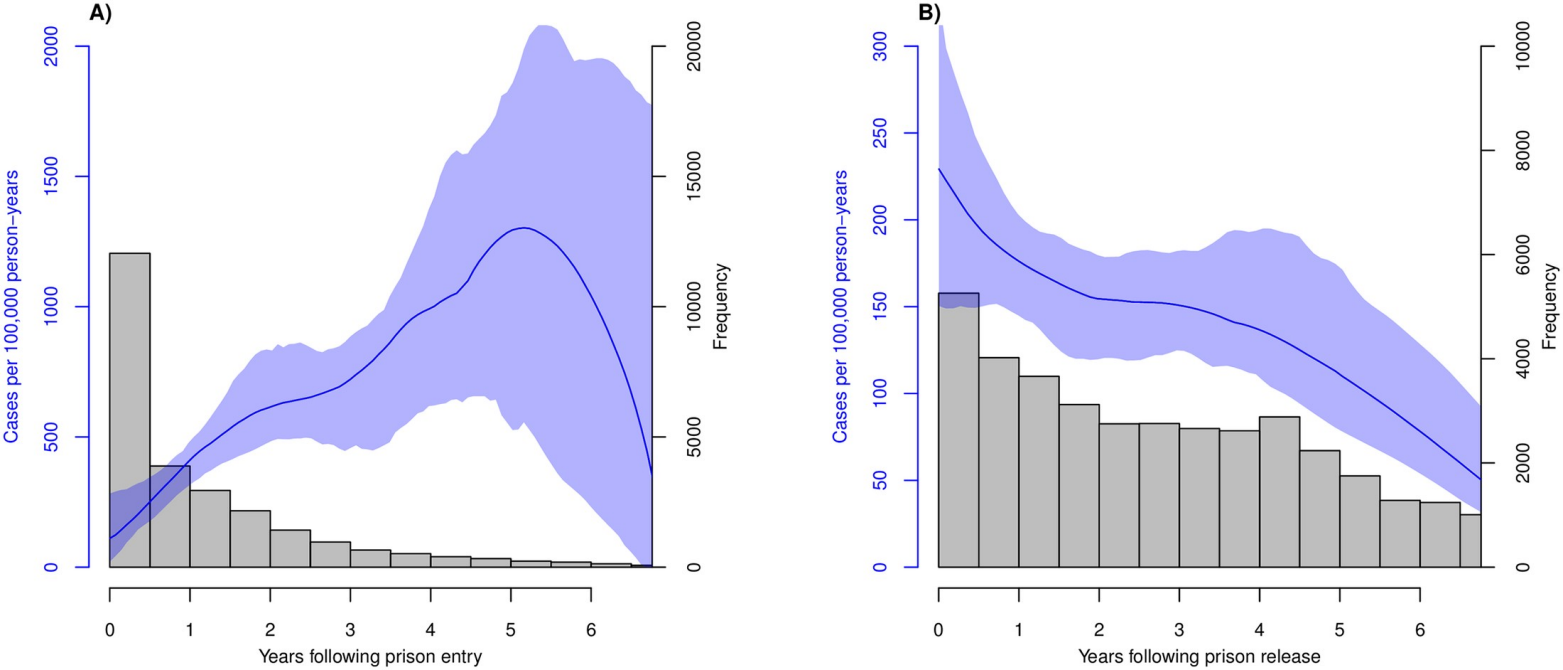
Incidence of TB among (A) Mato Grosso do Sul prisoners and (B) ex-prisoners based on length of incarceration and length of time following incarceration, respectively, with 95% bootstrap confidence intervals in blue shading. Histograms of individuals included in each survival analysis are overlaid. TB, tuberculosis.

For prisoners, we initiated follow-up at the start of first imprisonment and censored individuals at the time of release, death, or the end of the study period. For ex-prisoners, we began follow-up at the date of release from first incarceration and censored individuals at the time of reincarceration (if they had multiple incarcerations), death, or the end of the study period. All statistical analyses were performed in R version 3.5.0 [21]. We performed right-censored survival analyses for the prisoner and ex-prisoner cohorts using the package “survival” [22] and generated smoothed estimates of the hazard functions using the package “muhaz” [23]. We constructed 95% confidence intervals for hazard function estimates with 10,000 iterations of bootstrap resampling with replacement (Fig 2). The incidence of TB was calculated for both prisoners and ex-prisoners throughout the study period as the number of new cases every 6 months divided by the number of individuals at risk at the start of that time period and was then doubled to annualize rates.

We performed multivariate Cox regression upon the ex-prisoner cohort to identify any significant differences in active TB hazard between individuals who were in male or female prisons, within the largest maximum security prison or any other prison, or based on the duration of first incarceration. We evaluated the proportional hazards assumption through both graphical and statistical assessment of Schoenfeld residuals.

### Model

**Fig 3:**
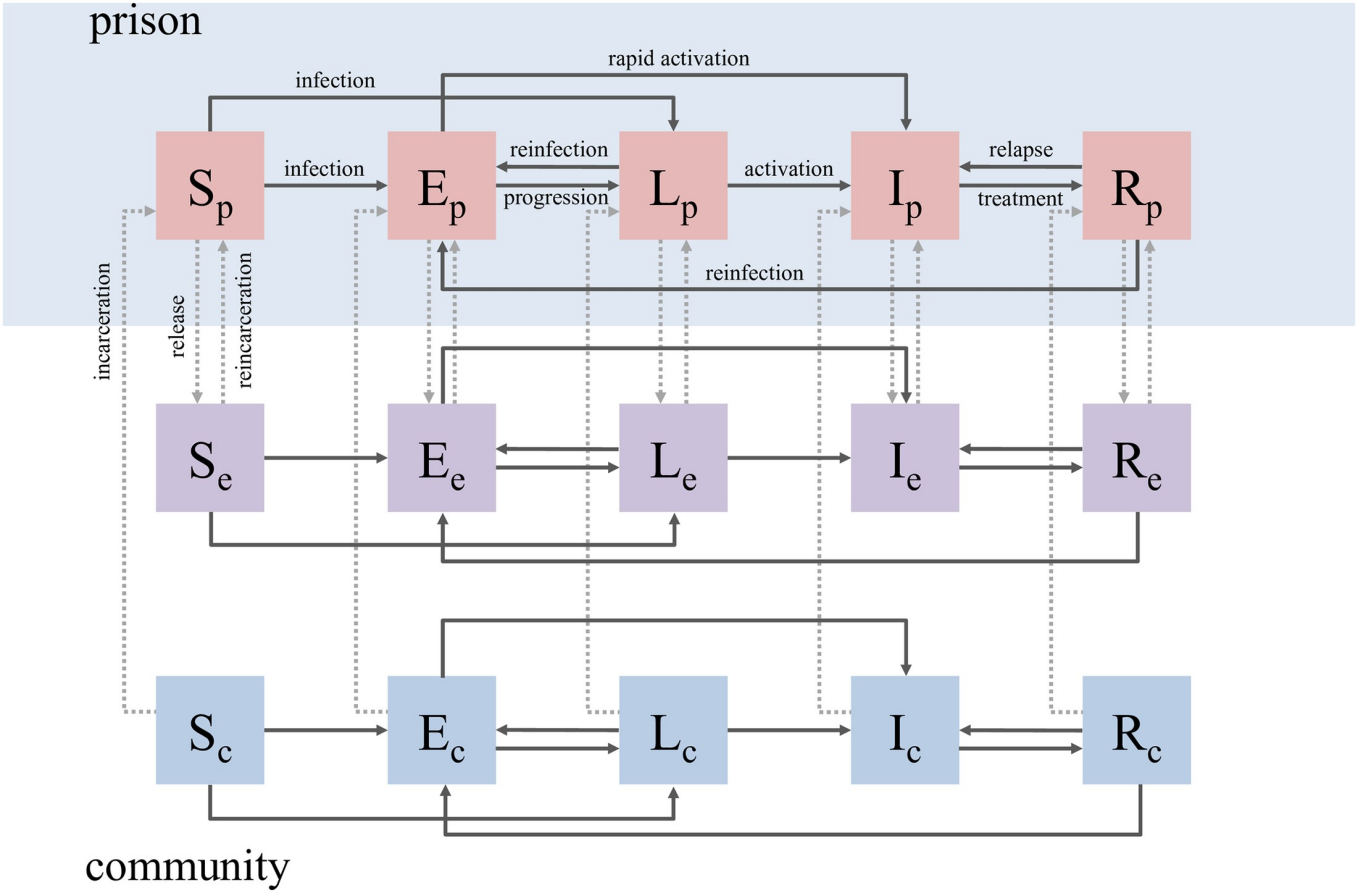
Compartmental model of TB transmission, describing the progression of disease from susceptible (S) to early latent infection (E), late latent infection (L), infectious (I), and recovery (R) among prisoners (subscript p, pink boxes), ex-prisoners (subscript e, purple boxes), and other community members (subscript c, blue boxes). Solid arrows represent the dynamics of TB transmission, whereas dotted arrows represent the dynamics of incarceration and release from prison. The prison environment is shaded blue to distinguish it from the external environment, which comprises ex-prisoners and other community members. TB, tuberculosis.

We created a mathematical model to simulate TB transmission dynamics among prisoners, ex-prisoners, and the general population. The model describes the prison population and corresponding local general population using a compartmental approach (Fig 3), characterizing the transitions between disease states, and linking the distinct populations of community members, prisoners, and ex-prisoners. Susceptible individuals (S), upon infection, move to an early latent stage (E) from which they can progress either directly (i.e., fast progression) to an infectious stage (I) or to a late latent stage (L). Late latent individuals may also progress to become infectious, and infectious individuals are diagnosed, rendered noninfectious by treatment, and considered recovered (R). Susceptible individuals are introduced into the population via birth (*μ*) and die at an equivalent rate in this closed-population model. Susceptible individuals are infected to the early latent stage with a force of infection equal to the transmission parameter (*β*) multiplied by the proportion of infectious individuals (I/N). Those with early latent infections progress to late latency or infectiousness at rates *w* and *τ*, respectively. Late latent individuals progress to the infectious stage at rate *v*, whereas infectious individuals are diagnosed and are immediately assumed to be started on therapy and no longer infectious at rate *d*. Individuals can be reinfected while in the late latent state (at rate *α*_*1*_ times the force of infection) or recovered state (at rate *α*_*2*_ times the force of infection) back to the early latent stage, which has a higher rate of progression to infectiousness. Recovered individuals are also susceptible to disease relapse at rate *γ*. Parallel SELIR states exist for prisoners, ex-prisoners, and never-incarcerated community members.

We parameterized the model with a combination of local data, well-characterized values from literature, and fitted values; as the factors driving TB transmission dynamics differ in the prison and community settings, the parameter values differ between the three population groups modeled (Table 1). We did not model multidrug-resistant (MDR) TB or HIV, as rates of both are low (<1% and <2%, respectively) in this context [24].

**Table 1:** Parameters used to describe TB natural history and epidemiology in a Brazilian state.

Variable	Definition	Prisoner	Ex-prisoner	Community	Source
*β*	effective contact rate	17.0 (10–30)	8.08 (4–12)	8.08 (4–12)	Calculated
*μ*	birth/death rate	0.017	0.017	0.017	[25]
*τ*	rate of progression from early latent to infectious	0.09 (0.005–0.1)	0.03 (0.005–0.1)	0.01 (0.005–0.1)	Calibration
*v*	rate of progression from late latent to infectious	.0008	.0008	.0008	[26]
*w*	rate of progression from early latent to late latent	0.2	0.2	0.2	[27]
*α*_*1*_	susceptibility to reinfection upon reexposure	0.21	0.21	0.21	[28]
*α*_*2*_	rate of reinfection from recovered to early latent	0.21	0.21	0.21	[28]
*γ*	rate of relapse from recovered to infectious	0.01	0.01	0.01	[29]
*d*	rate of diagnosis and treatment initiation	0.55 (0.5–1.0)	0.85	0.85	Calibration
*q*	rate of incarceration	N/A	0.0182	0.00196	Calculated
*r*	rate of release from imprisonment	0.5	N/A	N/A	Estimated from SIGO

Parenthetical values are the ranges used in sensitivity and uncertainty analyses. All rates are annual.

Abbreviations: SIGO, Sistema Integrado de Gestão Operacional; TB, tuberculosis.

For the rates of TB progression among prisoners and case detection, which are poorly characterized in the literature, we applied Latin Hypercube Sampling [30] to sample from distributions of values supported by the literature and a regularized sum-squared error process to select the sets that generated model incidences that best fit our prisoner and ex-prisoner incidence estimates. The model equations, calibration process, and parameter fitting methods are further described in the Supporting Information (S1 Text).

### Intervention strategies

We investigated the effects of prison-based interventions on the incidence of active TB after 10 years with our compartmental model of TB transmission. We considered the effect of a 25% improvement in the passive diagnosis rate, as may be achievable through replacement of sputum smear microscopy with rapid molecular diagnostics and chest radiography. We also investigated an active case detection intervention, whereby on a yearly basis, all individuals in the prisons are screened for active TB, regardless of whether they are symptomatic, and are appropriately placed on therapy if found to have disease. We then tested an entry screening program, in which all individuals entering prison are screened for active TB. Similarly, we tested an exit screening program. Finally, we modeled the provision of IPT, which aims to prevent disease by screening individuals who enter the prison for latent TB and providing IPT if they are found to be latently infected. Assumptions concerning the sensitivity, yield, and efficacy of these interventions are shown in the Supporting Information (S1 Table). Uncertainty analyses were performed using Latin Hypercube Sampling using the 100 best-fitting parameter sets from model calibration.

### Sensitivity analysis

We generalized our model of TB transmission in Mato Grosso do Sul to a broader range of epidemiologic scenarios in which the incarcerated population size and the relative incidence of disease were varied across a range of values observed in a recent meta-analysis [3]. We further varied the rate of assortative mixing, modeled as the relative rate of mixing among ex-prisoners and the general population, compared with their rate of mixing between the groups. For these general models, we assumed that the overall active TB incidence was the global average incidence (140 per 100,000 person-years) [12].

### Ethics statement

This study was approved by the following bodies: (1) the Stanford University Institutional Review Board (IRB-34301), (2) the Federal University of Grande Dourados (Opinion Number 877294), and (3) Agencia Estadual de Administracao do Sistema Penitenciario (signed document from the director of the state prison agency).

## Results

### TB incidence among prisoners and ex-prisoners

Between 2007 and 2013, 25,939 individuals were incarcerated for the first time in Mato Grosso do Sul, with a median duration of incarceration of 1.53 years (interquartile range [IQR]: 0.64–2.68). Among these individuals, we identified and verified 130 new active TB cases diagnosed during incarcerations. Among 38,241 inmates released from prison for the first time during this period, we verified 170 new cases (Fig 1). Median follow-up after release was 2.56 years (IQR: 1.04–4.31). For prisoners, active TB incidence increased from 111 per 100,000 person-years at the time of incarceration to a peak of 1,303 per 100,000 person-years at 5.2 years, after which it declined. Incidence at the median time of release (1.53 years) was 541 per 100,000 person-years. For ex-prisoners, active TB incidence was 229 cases per 100,000 person-years at release, declining to the baseline community incidence [12], 42 cases per 100,000 person-years, at slightly over 7 years (7.04 years) following release (Fig 2).

In Cox regression, the hazard of TB following release was 32% higher for each additional year of incarceration (adjusted hazard ratio [AHR]: 1.32, 95% BCI 1.19–1.48, *p* < 0.0001). The largest maximum security prison in the state did not have a significantly higher AHR for active TB upon release than all other prisons (AHR 1.65, 95% BCI 0.93–2.91, *p* = 0.09). However, the hazard for active TB upon release from female prisons was significantly lower than for male prisons (AHR 0.49, 95% BCI 0.29–0.84, *p* = 0.009). The proportional hazards assumption was tested by evaluating the relationship between Schoenfeld residuals and time for each individual covariate, which was nonsignificant (*p* = 0.86 for global test).

### Impact of interventions on TB incidence

**Fig 4:**
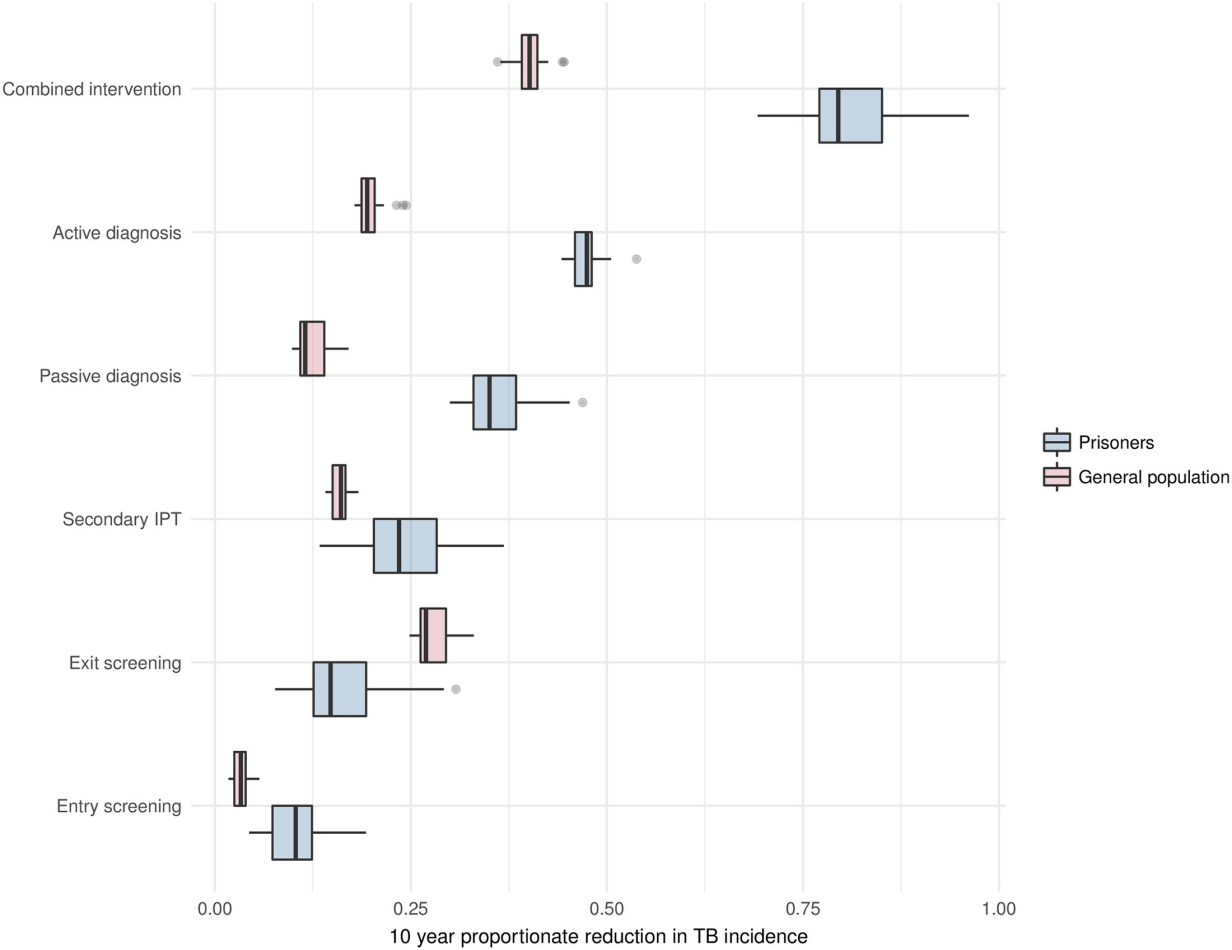
Proportionate decrease in TB incidence over 10 years according to various prison-based interventions. Box and whisker plots describe uncertainty in intervention effectiveness produced by Latin Hypercube Sampling analysis; boxes characterize 25th, 50th, and 75th percentile values; whiskers characterize a range of values up to 1.5 times the IQR; and dots represent outliers beyond this range. IPT, isoniazid preventive therapy; IQR, interquartile range; TB, tuberculosis.

We calibrated our model to contemporary estimates of baseline active TB prevalence in prisons (2,400 per 100,000) and incidence in the community (42 per 100,000). We then determined the effects of various interventions over a 10-year period, which are reported as relative incidence reductions and associated 95% Bayesian credible intervals (BCIs) (Fig 4).

If all individuals entering prisons were screened for active TB with 75% sensitivity, disease incidence would decrease in prisons by 10.3% (95% BCI 5.2%–18.8%) and by 3.3% (95% BCI 1.9%–5.6%) outside. If all individuals exiting prisons were screened, incidence would decrease in prisons by 14.8% (95% BCI 8.2%–30.0%) and by 27.0% (95% BCI 25.0%–32.4%) outside. Provision of isoniazid to those with latent TB at prison entry would reduce incidence in prisons by 23.5% (95% BCI 14.8%–36.8%) and by 16.1% (95% BCI 14.2%–18.2%) outside. Improvement in the passive case detection rate by 25% would decrease active TB incidence in prisons by 35.0% (95% BCI 30.1%–46.1%) and by 11.5% (95% BCI 10.0%–16.7%) outside. The single most effective intervention implemented was an annual active case detection campaign; this program would reduce active TB incidence in prisons by 47.4% (95% BCI 44.4%–52.2%) and by 19.4% (95% BCI 17.9%–24.2%) outside.

Combining these five approaches would decrease active TB incidence in prisons by 79.2% (95% BCI 70.2%–95.6%) and by 40.0% (95% BCI 36.1%–44.3%) outside.

### Sensitivity analyses

**Fig 5:**
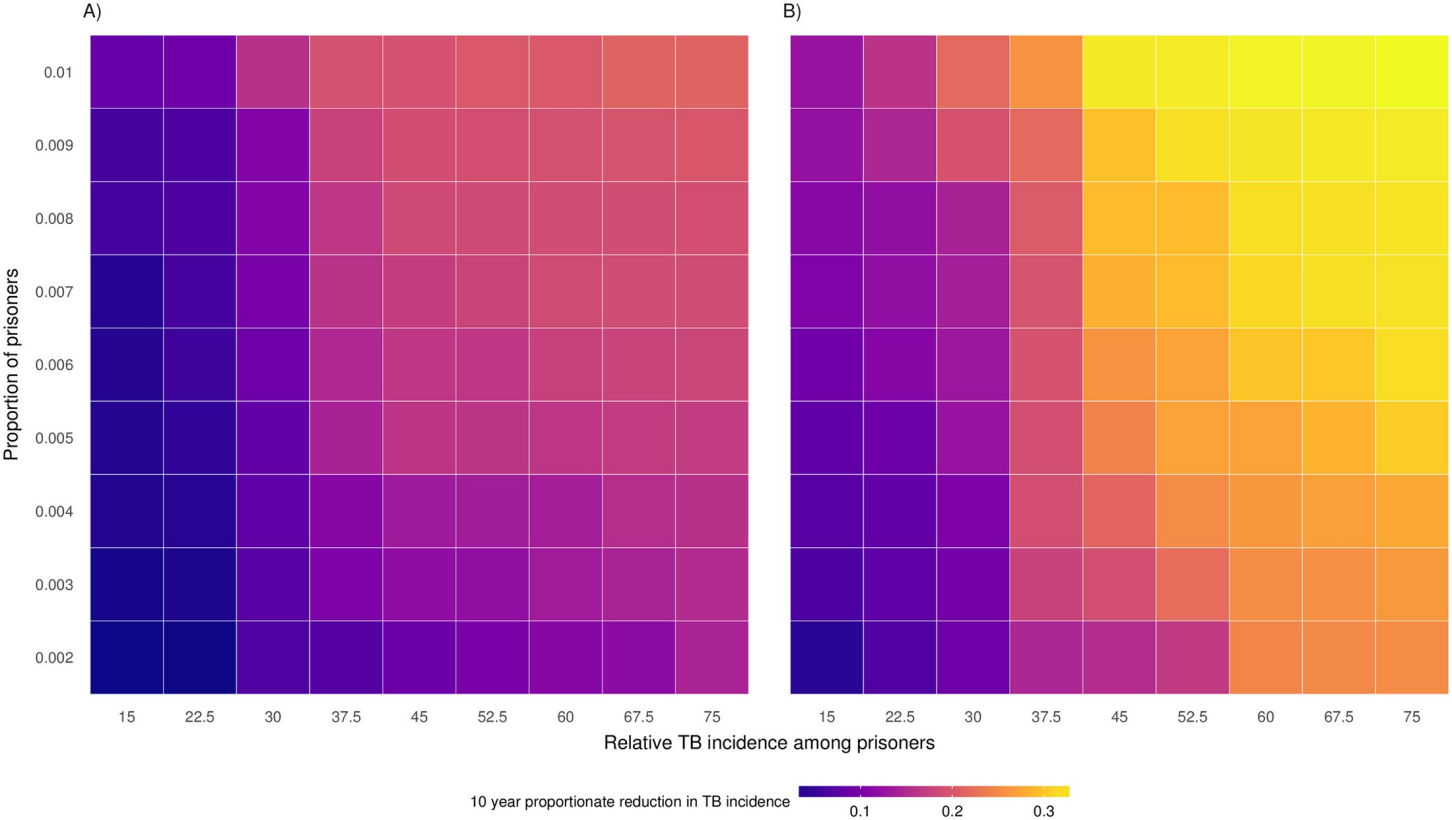
Heatmap of percent decrease in community TB incidence brought about by active diagnosis intervention across ranges of relative TB incidence (prisoner/community) and proportion of population incarcerated. Assumptions include (A) assortative mixing (3-fold relative rate of contact) for community members and ex-prisoners and (B) proportionate mixing between community members and ex-prisoners. TB, tuberculosis.

The relative incidence of active TB among prisoners and the proportion of prisoners in a population were both strongly correlated with the effectiveness of a prison-based active diagnosis intervention at the community level (Fig 5). The intervention achieved maximum effectiveness in a setting where there was a high proportion of incarcerated individuals (1% of the population) and a high relative incidence of active TB in the prisons compared to the community (a ratio of 75×). Conversely, the percent decrease in general population TB incidence was at a minimum when these two factors were the lower ends of the tested ranges (0.2% of population incarcerated and 15× relative incidence). When we modify the assumption of proportionate mixing between ex-prisoners and other community members to an assortative mixing pattern whereby each group is three times more likely to contact individuals within their own group, the magnitude of the intervention’s effectiveness remained considerable but was lower (Fig 5).

## Discussion

There is a critical need to develop new TB control strategies to achieve WHO’s global targets for 2035. One potential approach involves identifying and targeting “institutional amplifiers” or “reservoirs” of TB. We found that prisons in Brazil produce conditions favorable for amplifying TB epidemics in the general population. Incarcerated individuals enter prison with a low risk of TB, which increases rapidly over the course of 5 years, peaking at over 1,300 cases per 100,000 person-years, or 30 times higher than that of the general population. Upon release, former inmates have a nearly 5.5-times-greater risk of active TB than the general population—a risk that remains elevated for 7 years and is likely underestimated because of outmigration. Focusing interventions on prisons may therefore have potent effects on reducing rates of TB in the general population. We found that focusing interventions on prisoners, who constitute less than 0.5% of the population, would reduce the total active TB incidence in the population by over 40%. The avertable TB burden is greater than the proportion of cases that occur among prisoners, because of the high turnover of this population and dynamics of transmission in prisons and between prisons and communities. These findings underscore the importance of identifying and characterizing high-risk subpopulations for targeting TB control efforts.

A key question is whether the prison environment is the primary driver of TB risk or whether high TB rates in prisons are primarily a consequence of a population with preexisting high-risk attributes (e.g., HIV, substance abuse). The marked increase in TB risk that occurs upon incarceration, along with the decline in incidence following release, implicates the prison environment in driving disease in the population, whether it is due to transmission or acquired host factors (e.g., malnutrition, vitamin D deficiency) in the prison [31]. The observed peak of active TB incidence at 5 years and subsequent decline is consistent with an extremely high annual risk of infection occurring among a highly susceptible population, such that the majority of inmates would be exposed within 3–4 years. A recent study conducted in 12 prisons in Mato Grosso do Sul demonstrated that only 9% of inmates had latent TB at the time of incarceration, but an estimated 80% would be infected after 5 years [32]. The decline in active TB hazard after 5–6 years of incarceration could be explained by depletion of susceptible individuals—the majority of individuals have been infected and have either progressed to disease or established latency. However, we note that we had limited data from prisoners incarcerated over 5 years and caution against overinterpretation of this finding.

We find an increased rate of TB notifications among ex-prisoners, which declined steadily in the years following release but remained higher than that of the general population for several years. Given low transmission rates in the community, we believe this finding is driven primarily by infections acquired within prisons, which manifest in the years following release. Variability in disease progression and diagnosis results in a long declining tail of notifications following exit from the high-transmission prison environment [33]. Our model demonstrates that exit screening would reduce the burden of disease among ex-prisoners and therefore spillover of TB from prisons into the community. Exit screening could be even more effective in settings with low case-detection rates in prison.

This is the first analysis to model the impact of prison-focused interventions on preventing the spillover of TB into the general population. WHO recently led a systematic review of the evidence on TB case finding, but the panel was unable to reach consensus on active screening among prisoners, citing very low-quality evidence [34]. The conditional recommendation that it “should be considered” has not led to widespread implementation among LMICs because of the resources and high number of tests typically required to identify a case of active TB [35,36]. Of the interventions modeled, we found active screening within prisons to be the single most effective strategy in reducing TB incidence. Whereas passive case detection, provision of IPT, entry screening, and exit screening would have a more modest impact individually, a combined intervention including all of these approaches, together with mass screening, could avert nearly 80% of active TB cases in prisons and 40% of cases in the general population. Any public health interventions directed toward prisons must consider the unique risks faced by prisoners, and care must be taken in the design and implementation of interventions to ensure that human rights are protected. Interventions should be designed to benefit prisoners’ health, with community benefit as a secondary consideration.

Control programs often focus on entry screening in prisons, but our findings suggest that exit screening could also play an important role in reducing TB burden among prisoners at release and preventing population spillover; we believe this should be considered as a possible addition to guidelines. A three-phase program of screening at entry, periodically during incarceration, and upon exit has been utilized successfully in Malawi and may serve as a model [37].

To assess the generalizability of our model findings to other settings, we examined the potential impact of interventions across a range of epidemiologic scenarios for which the concentration of a population’s TB within prisons (the relative incidence rate and the size of the incarcerated population) varied across a range seen in LMICs. As anticipated, the impact of targeted interventions is greater in the settings with a larger incarcerated population and greater disparities in incidence between prisons and the general population. One critical unknown is the extent to which former prisoners mix with the general population, for which data are not available. Higher levels of assortative (e.g., “like with like”) mixing among ex-prisoners would attenuate the impact of prison-targeted interventions. However, given that the majority of TB transmission does not occur between individuals known to one another [38–40], assortative mixing among ex-prisoners is not likely to significantly curtail transmission to the general population. Moreover, even with substantial assortative mixing, we find that interventions targeted to prisoners would have a substantial effect on the population TB epidemic.

Our study has several limitations. We used state-level administrative databases, which likely contain some degree of diagnostic or demographic misclassification. To assess the hazard of primary TB, we limited our analysis among prisoners and ex-prisoners to individuals free of TB at baseline. This was done by filtering out individuals who were noted to have an episode of active TB prior to ever entering the prison system. However, we acknowledge that some individuals may have had prior TB that was not captured in SINAN, and so some of these primary TB cases may actually be recurrent disease. We only assessed TB hazard for individuals who were incarcerated for the first time, to mitigate analytical complexity; the duration and frequency of recurrent incarcerations vary widely, and the interpretation of TB risk across such variegated prisoner experiences would be difficult. As such, our estimates of TB hazard only apply to individuals who are newly incarcerated. However, our TB transmission model indeed includes recidivism and multiple incarcerations in order to accurately project the effect of interventions targeted to prisoners.

We were also unable to identify TB cases that occurred among ex-prisoners who left the state, as we only had TB data for the state of Mato Grosso do Sul. As a result, our findings concerning elevated TB incidence among former prisoners are conservative, since they exclude TB cases that may have occurred in other states. In a recent study of 13 prisons in Mato Grosso do Sul, 36% of prison inmates reported being from another state in Brazil [14]. These data suggest that a significant portion of the prison population may leave the state following release, and so TB incidence among ex-prisoners may be considerably higher than we have calculated. We assumed that the TB epidemic in the community and prisons is at steady state, and population-level incidence has changed by <5% in the past 15 years; if TB incidence disparities are growing, as some evidence suggests [17], our model would underestimate the benefits of interventions targeted in the prisons. Our model is a simplification of a complex process; we deliberately chose a parsimonious model structure that builds directly upon widely used models in the TB literature. Although more complex models would enable better fit to the local data [41], they would risk overfitting and make it difficult to generalize results to other epidemiologic contexts.

Globally, more than 10 million individuals are incarcerated, an increase of 20% since 2000 [13]. Virtually all studies of TB incidence in prisons have found elevated risk compared to the general population, with incidence rate ratios ranging from 2.5 to 151.1 [3]. Our data suggest that prisons represent a major source of TB transmission that undermines population-level TB control. The risk factors intrinsic to prison environments, more so than inherently high-risk prison populations, drive TB incidence, and targeted interventions within prisons could have a substantial effect on the broader TB epidemic.

## Supporting information

S1 Text Description of equations, assumptions, calibration process, intervention scenarios, and sensitivity analyses applied to model of TB transmission dynamics. TB, tuberculosis.
(DOCX)

S1 Table Summary of prison-based interventions implemented in the model. (DOCX)

S1 STROBE STROBE Checklist. (DOC)

## Abbreviations

AHR: adjusted hazard ratio
BCI: Bayesian credible interval
IPT: isoniazid preventive therapy
IQR: interquartile range
LMIC: low- and middle-income country
MDR: multidrug-resistant
SIGO: Sistema Integrado de Gestão Operacional
SINAN: Sistema de Informação de Agravos de Notificação
TB: tuberculosis
WHO: World Health Organization
