# Correction: Evaluating strategies for control of tuberculosis in prisons and prevention of spillover into communities: An observational and modeling study from Brazil

**DOI:** 10.1371/journal.pmed.1002764

**Published:** 2019-03-01

**Authors:** Tarub S. Mabud, Maria de Lourdes Delgado Alves, Albert I. Ko, Sanjay Basu, Katharine S. Walter, Ted Cohen, Barun Mathema, Caroline Colijn, Everton Lemos, Julio Croda, Jason R. Andrews

In the Funding section, the first grant number from the funder National Institutes of Health is listed incorrectly. The correct grant number is: #R01 AI130058.

## References

[1] Mabud TS, de Lourdes Delgado Alves M, Ko AI, Basu S, Walter KS, Cohen T, et al (2019) Evaluating strategies for control of tuberculosis in prisons and prevention of spillover into communities: An observational and modeling study from Brazil. PLoS Med 16(1): e1002737 10.1371/journal.pmed.1002737 30677013 PMC6345418

