# Supplementary material for: Evaluating strategies for control of tuberculosis in prisons and prevention of spillover into communities: An observational and modeling study from Brazil
Source: PLoS Med. 2019 Jan 24;16(1):e1002737. doi: 10.1371/journal.pmed.1002737 (PMC6345418; doi:10.1371/journal.pmed.1002737)
Supplement: S1 Text — TB, tuberculosis. (DOCX) [file pmed.1002737.s001.docx]

# **Supporting Information**

*Model equations and assumptions*

We adapted widely used compartmental TB natural history and transmission models, governed by a system of ordinary differential equations, to describe TB transmission dynamics in a Brazilian state, among three groups: prisoners, ex-prisoners, and community members (Figure 3). We assume no HIV, no MDR TB, and a single strain model. In this setting, MDR TB is rare (<1%) and HIV prevalence is <2%, and are therefore unlikely to alter model dynamics [1]. The subscripts p, e, and c in the following equations and text denote prisoners, ex-prisoners, and community members, respectively.

The transmission parameter, β was calculated for each population group in the model using the following formula,

Equation 1:

β = λ/I

where λ is equivalent to the force of infection, and *I* can be estimated as the prevalence of TB disease. For prisoners, both the force of infection for prisoners and the TB prevalence were obtained from a recent prospective study of TB infection in Mato Grosso do Sul prisons [2], yielding a β_P_ of 17. For community members and ex-prisoners, λ was estimated using the following formula,

Equation 2:

λ = $-\frac{1}{t}ln(1-proportion infected)$

where *t* is time. In this equation, *t* is set to 25 years, the average age of first incarceration, and cumulative risk of infection at age 25 is 10%, yielding a force of infection of 0.0042. With the prevalence of TB disease in Brazil obtained from WHO surveillance data [3], it is possible to then solve for β.

Because prisoners, ex-prisoners, and community members can all experience the same disease states, the set of differential equations for these three groups is largely identical. The equations differ in that ex-prisoners and community members can be incarcerated at rate *q*, while prisoners can be released at rate *r*. Additionally, while prisoners are only infected (and reinfected) at a rate proportional to the imprisoned infectious population, ex-prisoners and community members are susceptible to infection (and reinfection) through contact with any infectious individual outside of the prison.

Annual release rates were derived from the SIGO report. We solved the following system of equations using known values from Mato Grosso do Sul to determine the incarceration rates for community members and ex-prisoners (*q*_c_ and *q_e_*) at model equilibrium:

Equation 3:

dC/dt = *μ*N - *q*_c_C - *μ*C

dP/dt = *q*_c_C + *q*_e_E - *r*P - *μ*P

dE/dt = *r*P - *q*_e_E - *μ*E

Individuals are born into the model as either susceptible, early latent, or late latent community members, at a rate proportional to the overall population of the model. Birth and death are equilibrated to maintain a fixed population. Upon introduction into the model, individuals are assumed to be adults and of incarcerable age (15 years old). The proportion of susceptible, early latent, and late latent individuals born into the population (at age 15) are governed by the following exponential equations:

Equation 4:

Proportion S = 1-(1-e^-rt^)

Proportion E = ⅓(1-e^-rt^)

Proportion L = ⅔(1-e^-rt^)

Here, the rate (r) is equivalent to the per capita force of infection for a community member, *β*(I_c_/N_c_), and the time (t) is 15 years. We assume that the ratio of late latent to early latent individuals born into the population at age 15 is 2:1.

We structured the model such that the total population is equal to 1, and each compartment in the model is a proportion. For both the infectious and susceptible individuals at the initiation of the model, we assumed that 0.5% were prisoners, 0.5% were ex-prisoners and 99% were community members, and brought it to equilibrium as governed by incarceration, release, birth and death rates.

We assumed proportionate mixing between community members and ex-prisoners in our assessment of TB transmission, although we explored an assortative mixing pattern in our sensitivity analysis.

Equation 5.

N_p_ = S_p_+E_p_+L_p_+I_p_+R_p_

N_e_ = S_e_+E_e_+L_e_+I_e_+R_e_

N_c_ = S_c_+E_c_+L_c_+I_c_+R_c_

*Prisoners*

dS_p_/dt = -β_p_(I_p_/N_p_)S_p_ - μ_p_S_p_ + *q*_c_S_c_ + *q*_e_S_e_ - *r*S_p_

dE_p_/dt = β_p_(I_p_/N_p_)S_p_ - *w*_p_E_p_ - *τ*_p_E_p_ - μ_p_E_p_ + *q*_c_E_c_ + *q*_e_E_e_ - *r*E_p_ + α_1_β_p_(I_p_/N_p_)L_p_ + α_2_R_p_

dL_p_/dt = *w*_p_E_p_ - *v*_p_L_p_ - μ_p_L_p_ +*q*_c_L_c_ + *q*_e_L_e_ - *r*L_p_ - α_1_β_p_(I_p_/N_p_)L_p_

dI_p_/dt = *τ*_p_E_p_ + *v*_p_L_p_ - *d*_p_I_p_ - μ_p_I_p_ +*q*_c_I_c_ +*q*_e_I_e_ + γR_p_ - *r*I_p_

dR_p_/dt = *d*_p_I_p_ - μ_p_R_p_ + *q*_c_R_c_ + *q*_e_R_e_ - *r*R_p_ - α_2_R_p_ - γR_p_ +*q*_c_I_c_ +*q*_e_I_e_

*Ex-prisoners*

dS_e_/dt = -β_e_(I_e_/(N_e_+N_c_))S_e_ - β_c_(I_c_/(N_c_+N_e_))S_e_ - μ_e_S_e_ + *r*S_p_ -*q*_e_S_e_

dE_e_/dt = β_e_(I_e_/(N_e_+N_c_))S_e_ + β_c_(I_c_/(N_c_+N_e_))S_e_ - *w*_e_E_e_ - *τ*_e_E_e_ - μ_e_E_e_ + *r*E_p_ - *q*_e_E_e_ + α_1_(β_e_(I_e_/(N_e_+N_c_))L_e_ + β_c_(I_c_/(N_c_+N_e_))L_e_) + α_2_R_e_

dL_e_/dt = *w*_e_E_e_ - *v*_e_L_e_ - μ_e_L_e_ + *r*L_p_ - *q*_e_L_e_ - α_1_(β_e_(I_e_/(N_e_+N_c_))L_e_ + β_c_(I_c_/(N_c_+N_e_))L_e_)

dI_e_/dt = *τ*_e_E_e_ + *v*_e_L_e_ - *d*_e_I_e_ - μ_e_I_e_ + *r*I_p_ + γR_e_ - *q*_e_I_e_

dR_e_/dt = *d*_e_I_e_ - μ_e_R_e_ + *r*R_p_ - *q*_e_R_e_ - α_2_R_e_ - γR_e_

*Community*

dS_c_/dt = ((1-(1-*e*^(-βc(Ic/Nc)15)^))μN - β_e_(I_c_/(N_c_+N_e_))S_c_ - β_c_(I_e_/(N_e_+N_c_))S_c_ - μ_c_S_c_ -*q*_c_S_c_

dE_c_/dt = (1/3)(1-*e*^(-βc(Ic/Nc)15)^)μN + β_e_(I_c_/(N_c_+N_e_))S_c_ + β_c_(I_e_/(N_e_+N_c_))S_c_ - *w*_c_E_c_ - *τ*_c_E_c_ - μ_c_E_c_ - *q*_c_E_c_ +

α_1_(β_e_(I_c_/(N_c_+N_e_))L_c_ + β_c_(I_e_/(N_e_+N_c_))L_c_) + α_2_R_c_

dL_c_/dt = (2/3)(1-*e*^(-βc(Ic/Nc)15)^)μN + *w*_c_E_c_ - *v*_c_L_c_ - μ_c_L_c_ - *q*_c_L_c_ - α_1_(β_e_(I_c_/(N_c_+N_e_))L_c_ + β_c_(I_e_/(N_e_+N_c_))L_c_)

dI_c_/dt = *τ*_c_E_c_ + *v*_c_L_c_ - *d*_c_I_c_ - μ_c_I_c_ + γR_c_ - *q*_c_I_c_

dR_c_/dt = *d*_c_I_c_ - μ_c_R_c_ - *q*_c_R_c_ - α_2_R_c_ - γR_c_

*Model calibration*

We calibrated the model to our calculated TB incidence data from Mato Grosso do Sul. We fitted only a small set of model parameters which was not well characterized by past studies or our data (Table 1); attempting to fit all of the parameters in our model would generate specious values which would nicely fit data points, but lack real-world validity [9]. Parameter fitting allowed us to characterize epidemiological parameters for which there is currently a poor understanding, such as the rate of TB progression from high-risk early latency to lower-risk late latency among prison inmates, as well as the rate of TB diagnosis in the prisons.

To account for uncertainty in parameter selection, Latin Hypercube Sampling was used to create 1000 parameter sets of *τ*, *v*, and *d*. The hypercube was populated by drawing from uniform distributions containing plausible parameter values, shown in parentheses in Table 1 for the relevant parameters. We then calculated the sum squared error of each parameter set using the following equations:

Equation 6:

A = Sum squared error prisoners= Sum(Calculated incidence - model incidence)^2^

B = Sum squared error ex-prisoners and community = X*Sum(Calculated incidence - mode incidence)^2^

Combined sum squared error = A+B

Here, X is a regularization parameter equal to the calculated incidence among prisoners divided by the combined calculated incidence among community members and ex-prisoners, introduced to prevent overfitting to the prisoner incidence. We ordered the parameter sets from lowest to highest sum squared error terms.

*Intervention scenarios*

Various administrative interventions were implemented in the model, as described in S1 Table, to explore the effects of prison-based intervention on community TB burden. Each of the interventions was applied after the base model reached equilibrium, and each intervention was run for a period of 10 years.

For the isoniazid preventive therapy intervention, two separate compartments were introduced into the model. As individuals enter the prison, they are screened for latent TB with a sensitivity of 0.79 [10]. If they are found to have latent TB, they are voluntarily started on IPT with an assumed treatment acceptance rate of 75%, which is represented by a compartment P, for a period of 6 months. During this time, the individuals are essentially immune to TB infection. After the course of IPT, the individuals move from compartment P to compartment S_L_, representing the post-treatment group. These individuals are susceptible to TB reinfection, and can progress to E_p_ at rate *α_2_* times the force of infection among prisoners, as they (like late latent individuals) are presumed to have some immunity to re-infection. Normal rates of death and release from prison apply to compartments P and S_L_. However, prisoners released from P are released to E_e_ and L_e_ (latent infections) proportional to their respective populations in the prison. Prisoners released from S_L_ become susceptible ex-prisoners (S_e_).

*Sensitivity analysis: relative incidence and proportion of prisoners*

Our sensitivity analysis broadens the generalizability of our TB model, by varying three different parameters: 1) the proportion of prisoners within the model community, 2) the relative incidence of TB in the prisons compared to the community, and 3) the mixing patterns of ex-prisoners and community members outside the prison.

We modified our original model of TB transmission by fixing all parameters (best fit values in Table 1) except the *β* terms, and fitting the combined prisoner/community model incidence to 140/100k (global median) [3]. Where pp is the proportion of prisoners, RI is the relative incidence, and I_c_ is the incidence in the community, we use the following formula to calculate a matrix of I_c_, for pp range (.002, .01) and RI range (15, 75):

Equation 7.

pp*RI*Ic + (1-pp)*Ic = 140/100k

The incidence in prisons (I_p_) is then calculated as the product of Ic and RI for each point in the matrix. Thereafter, the prevalence in the community (P_c_) and in prisons (P_p_) is calculated from each of the I_c_,I_p_ values, as the product of incidence and duration of infection (*d*).

We indirectly modify the population of prisoners in the model by modifying the community incarceration rate (*q*_c_). To do so, we solve the system of differential equations (Equation 3) of individual movement between the three model environments (community, prisoner, ex-prisoner) at steady state, for the values of *q*_c_ which produce the desired proportions of prisoners.

We then fit the model to each P_c_,P_p_ value set (with *β* terms fitted as described in Methods, and with *q*_c_ coming from the values solved as described), to get the model prevalence for the active diagnosis intervention, allowing us to calculate a percent decrease for each point on the pp vs RI matrix of P_c_,P_p_ values.

To assess intervention efficacy based on different mixing patterns for individuals in the community, we performed the above sensitivity analysis with two different assumptions: 1) ex-prisoners and community members mix proportionately outside the prison; 2) ex-prisoners and community members assortatively mix, such that they are three times more likely to interact with their own cohort than the other (Figure 5). To modify the mixing patterns in the model, the *β* terms were repurposed as follows. While *β*_p_ was not changed, the *β*_e_ and *β*_c_ terms were eliminated and replaced by *β*_1_ and *β*_2_ terms, where *β*_1_ was applied to terms in the model indicating contact was occurring among members of the same cohort (ex-prisoner or community), and *β*_2_ was applied to any term in the model indicating contact between the two cohorts. The *β*_p_, *β*_1_, and *β*_2_ terms were then fit using the same procedure described in methods, with the constraint that *β*_1_=*β*_2_ for the proportionate mixing assumption, and *β*_1_=3**β*_2_ for the assortative mixing assumption.

**References**

1. Sgarbi RVE, Da Silva Santos Carbone A, Paião DSG, Lemos EF, Simionatto S, Puga MAM, et al. A cross-sectional survey of HIV testing and prevalence in twelve brazilian correctional facilities. Yu X-F, editor. PLoS One. 2015;10: e0139487. doi:10.1371/journal.pone.0139487

2. Carbone A da SS, Paião DSG, Sgarbi RVE, Lemos EF, Cazanti RF, Ota MM, et al. Active and latent tuberculosis in Brazilian correctional facilities: A cross-sectional study. BMC Infect Dis. 2015;15: 24. doi:10.1186/s12879-015-0764-8

3. Organization WH. Global tuberculosis report 2013. World Health Organization; 2013.

4. Salomon JA, Wang H, Freeman MK, Vos T, Flaxman AD, Lopez AD, et al. Healthy life expectancy for 187 countries, 1990–2010: a systematic analysis for the Global Burden Disease Study 2010. Lancet. Elsevier; 2012;380: 2144–2162.

5. Shea KM, Kammerer JS, Winston CA, Navin TR, Horsburgh CR. Estimated rate of reactivation of latent tuberculosis infection in the United States, overall and by population subgroup. Am J Epidemiol. 2014;179: 216–225. doi:10.1093/aje/kwt246

6. Sutherland I, Švandová E, Radhakrishna S. The development of clinical tuberculosis following infection with tubercle bacilli. Tubercle. 1982;63: 255–268. doi:10.1016/S0041-3879(82)80013-5

7. Andrews JR, Noubary F, Walensky RP, Cerda R, Losina E, Horsburgh CR. Risk of progression to active tuberculosis following reinfection with Mycobacterium tuberculosis. Clin Infect Dis. Oxford University Press; 2012;54: 784–791. doi:10.1093/cid/cir951

8. Blower SM, McLean AR, Porco TC, Small PM, Hopewell PC, Sanchez MA, et al. The intrinsic transmission dynamics of tuberculosis epidemics. Nat Med. 1995;1: 815–821. doi:10.1038/nm0895-815

9. Jacquez JA. Identifiability and model distinguishability. Compart Anal Biol Med. 1985; 309–354.

10. Kahwati LC, Feltner C, Halpern M, Woodell CL, Boland E, Amick HR, et al. Primary care screening and treatment for latent tuberculosis infection in adults: Evidence report and systematic review for the US Preventive Services Task Force [Internet]. JAMA - Journal of the American Medical Association. 2016. pp. 970–983. doi:10.1001/jama.2016.10357
