# Supplementary material for: Evaluating strategies for control of tuberculosis in prisons and prevention of spillover into communities: An observational and modeling study from Brazil
Source: PLoS Med. 2019 Jan 24;16(1):e1002737. doi: 10.1371/journal.pmed.1002737 (PMC6345418; doi:10.1371/journal.pmed.1002737)
Supplement: S1 Table — (DOCX) [file pmed.1002737.s002.docx]

**S1 Table:** Summary of prison-based interventions implemented in model

| Name | Description | Model Assumptions |
| --- | --- | --- |
| Entry screening | Proactive screening of all individuals newly incarcerated as they enter the prison | Actively infected individuals who are entering the prisons are sent directly to R_p_. The screening is assumed to be 75% sensitive, with the false negatives sent to I_p_. |
| Exit screening | Proactive screening of all individuals as they are released from prison | Actively infected individuals who are exiting the prisons are sent directly to R_e_. The screening is assumed to be 75% sensitive, with false negatives sent to I_e_. |
| Isoniazid preventive therapy | Starting latently infected individuals on isoniazid with the intent of preventing progression to infectiousness | Latently infected individuals entering prison are screened and those who accept treatment are put into P. After people go to P, they go to S_L_, from which they can be reinfected to E, but cannot go to I. |
| Improved passive diagnosis | Reactive screening of individuals for TB when they present with symptoms | Increase diagnosis rate (*d*) by 25%. |
| Active diagnosis | Proactive screening of incarcerated individuals for TB when they are asymptomatic | Increase instantaneous diagnosis rate (*d*) to infinity at the end of each year for 10 years |
